# A comparison of patients' perceptions and an audit of health promotion practice within a UK hospital

**DOI:** 10.1186/1471-2458-7-242

**Published:** 2007-09-13

**Authors:** Charlotte L Haynes, Gary A Cook

**Affiliations:** 1 Epidemiology, Stockport NHS Foundation Trust, Stepping Hill Hospital, Stockport SK2 7JE, UK

## Abstract

**Background:**

UK hospitals are required to monitor the health promotion services they provide for patients. We compared the use of audit and patient questionnaires as appropriate tools for monitoring whether patients are screened for modifiable risk factors (smoking, alcohol use, obesity, diet, and physical activity), whether staff correctly identify risk factor presence and deliver health promotion when a risk factor is identified.

**Methods:**

We sent a questionnaire post-discharge to 322 hospitalised adult patients discharged alive between January and March 2006, and audited their hospital case notes for evidence of screening for risk factors, identification of risk factors, and delivery of health promotion to change risk factors. Agreement between the audit and questionnaire findings was assessed by Kappa statistic.

**Results:**

There was little relationship between what was written in the case notes and what patients thought had happened. Agreement between the audit and questionnaire for screening of risk factors was at best fair. For the delivery of health promotion agreement was moderate for alcohol, poor for exercise, and no different from chance for smoking and diet. Agreement on identifying risk factors was very good for obesity, good for smoking, and moderate for alcohol misuse. The identification of diet quality and level of physical activity was too low in the audit to allow statistical comparison with self-reported diet and activity.

**Conclusion:**

A direct comparison of data gathered in the audit and patient questionnaires provides a comprehensive picture of health promotion practice within hospitals. Poor screening agreement is likely to be due to errors in patients' recall of screening activities. Audit is therefore the preferred method for evaluating screening of risk factors, but further insight into screening practice can be gained by using the questionnaire in conjunction with audit. If a patient does not recognise that they received health promotion, then this is tantamount to not receiving it, we therefore recommend that the patient questionnaire is the preferred method for monitoring health promotion delivered. For monitoring the accuracy of risk factor identification either method is appropriate as long as the hospital uses validated screening tools for identifying alcohol misuse, diet, and physical activity.

## Background

In England, health promotion, defined as *"the process of enabling people to increase control over, and to improve, their health" *[1] is a priority for every NHS organisation. The target areas are reducing smoking, tackling obesity (focusing on improving diets and increasing physical activity), sensible drinking, improving sexual health and mental health and tackling health inequalities [2]. Hospitals must, amongst other criteria, collect, analyse and provide data on the current and future health and healthcare needs of their local population [3]. They are required to provide or commission disease prevention/health promotion services and programmes, monitor these services/programmes, and use findings to inform the planning of further services/programmes [3].

**Figure 1:**
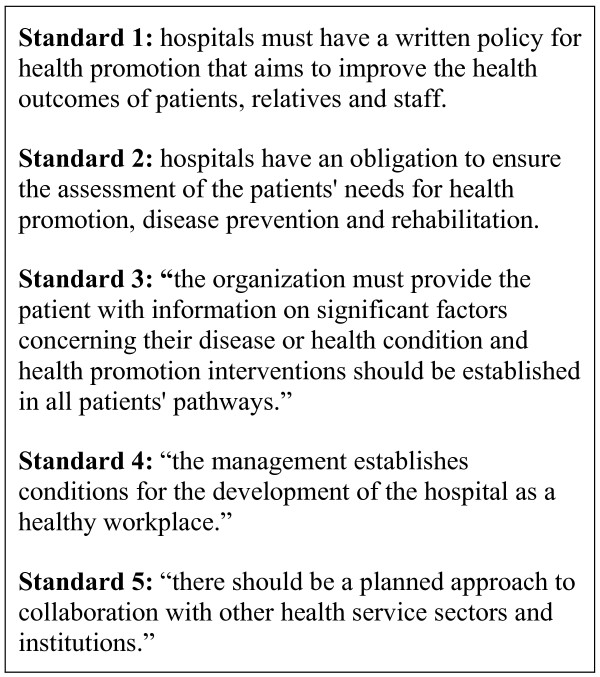
WHO standards for health promotion in hospitals [4].

The World Health Organisation (WHO) Network of Health Promoting Hospitals supports hospitals in developing and delivering health promotion services. It has set five standards and indicators to ensure the quality of health promotion services in hospitals (see figure 1) [4,5]. The standards state that all hospitals should have in place programmes for health promotion and disease prevention for patients, relatives and staff, and that a collaborative approach with other health services is required for successful delivery of health promotion.

**Figure 2:**
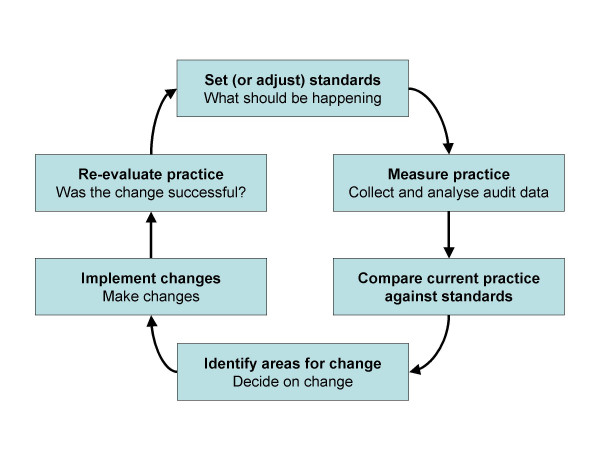
The Audit Cycle. Adapted from [6].

The question is how best to assess health promotion practice within a hospital. One informative method is audit. Clinical audit can be defined as "*a quality improvement process that seeks to improve patient care and outcomes through systematic review of care against explicit criteria and the implementation of change*" [6]. The audit cycle is described in figure 2. Commonly clinical audits within secondary care are based on data contained within patients' written case notes. While audit should be an objective way of measuring and monitoring practice against a set of agreed standards, there may be a mismatch between the written word and actual practice.

Several studies have compared medical records and/or patients' recall of health promotion with the "gold standard" of direct observation (researchers observing practice, audiotape or videotape) [7-10]. These studies have found that patients' recall of assessment of risk factors and health promotion delivered by healthcare professionals during consultations is more accurate than documentation in medical records [6,7]. For example, Wilson and Macdonald (1994) found that compared with audio-tape of 516 primary care consultations, medical records accurately reported provision of smoking advice and alcohol advice in only 28.6% and 31.1% of cases respectively, while patients' recall was accurate in 73.9% and 75% of cases respectively (compared to 355 audio-taped consultations) [7]. To our knowledge, these types of study have only been undertaken within primary care settings. There is one study comparing medical record documentation with patients' recall of a smoking cessation programme delivered in a US hospital, but the findings were not compared with a "gold standard" measure [11]. The findings do however corroborate findings within primary care: physicians documented smoking cessation counselling in only 46.2% of cases while patients reported it in 71.0% of cases.

We undertook an initial audit of health promotion practice for modifiable risk factors within one UK hospital [12], which appeared to show that whilst screening for smoking and alcohol use were performed in the majority of cases, health promotion was infrequently delivered. Moreover screening and delivery of health promotion for diet, exercise, and obesity were poor.

Given the evidence suggesting that medical records are not highly accurate for documentation of health promotion services, we felt that information gathered from written case notes might have been a poor reflection of actual health promotion practice. For example, health promotion might have been delivered but not described (resulting in false negatives); or the absence of a risk factor might not have been documented in case notes (i.e. someone is not a smoker, not obese, etc). Alternatively, fewer patients might in reality have been screened and received health promotion for risk factors than was indicated within case notes (false positives). To explore these hypotheses we undertook a second audit combined with a questionnaire to all patients whose case notes were included in the audit. This study is unique in that it directly compares medical records and patients' recall of health promotion delivered within a UK hospital, and that it investigates a wide range of services delivered through multiple contacts with healthcare professionals during routine hospital care.

## Methods

### Setting

The setting for this study was a District General Hospital located in the Northwest of England. At the time of this study the hospital did not have an explicit health promotion policy, but all patients were expected to be assessed for risk factors on admission or prior to surgery for elective surgery patients (as evidenced by various Integrated Care Pathway (ICP) documents). Training of staff in health promotion was patchy – some nurses were trained in smoking cessation – but there were some specialists (1 alcohol liaison nurse and several dieticians). Some ICP documents provided details of advice, appropriate leaflets and referral pathways for patients with identified risk factors.

### Participants

Participants were adult patients (≥ 17 years old) discharged alive from the hospital between January 1^st ^and March 31^st ^2006 from twelve surgical and medical wards. Participants had been in-patients or day-cases.

### Data collection: instruments and procedures

Case note numbers for all patients discharged alive between January and March 2006 were identified within one day of their discharge. Attempts were made to get all case notes and audit them to determine whether patients had been screened for smoking, alcohol use, diet, exercise, and weight, whether health promotion needs had been identified, and whether health promotion had been delivered. A research assistant either accessed case notes on wards (prior to being sent to clinical coding or the patient's consultant), from the hospital's medical library, or clinical coding department. Patients who were terminally ill were excluded from this audit.

Case notes for the last hospital episode only were audited (date of admission to date of discharge). Any mention within a patient's case notes that they were or were not a smoker was recorded as screening for smoking. A record of alcohol intake was classified as screening for alcohol. A record of body mass index (BMI = weight (Kg)/height (m)^2^) indicated that the patient had been screened for obesity. A BMI >25 is indicative of overweight and a BMI >30 indicates obesity. Screening of diet and exercise were more difficult to establish, so any written indication that the patient was asked about their normal diet and physical activity was taken to mean that they had been screened for diet and exercise respectively.

Health promotion was deemed as "needed" for each risk factor independently. That is, if someone was a smoker they should received health promotion for smoking cessation. For alcohol: consumption of alcohol above recommended weekly limits of greater than 21 units for men, and greater than 14 units for women. For weight management health promotion: (including diet and exercise) a BMI > 25 (individuals were further categorised into overweight and obese). For exercise: evidence of engaging in less than thirty minutes of moderate physical activity five times a week [2]. Guidelines set by the Department of Health concerning what a healthy diet should consist of, for example consuming five portions of fresh fruit and vegetables a day were used to establish whether an individual required health promotion for diet [2].

Any documentation that the patient had received verbal advice, literature or referral to a specialist for an identified risk factor was taken as indicative of health promotion delivered.

We sent a postal, self-completion questionnaire to all patients whose case notes were audited within one month of their discharge from hospital. We chose an upper limit of one month to minimise the chances of patients' forgetting about the healthcare they had received. The questionnaire followed the same format for each health-related behaviour – smoking, alcohol use, diet and weight, and exercise. First, patients were asked whether anyone at the hospital had assessed their risk factor status (screening). These questions took the form of "During your hospital admission were you asked about *risk factor*?" Participants could answer "yes" or "no". Second, information was gathered on the patient's behaviour in relation to each of the five areas. Smoking was assessed by 3 questions: "Do you currently smoke?" and "have you ever smoked?" (response: "yes" or "no"). Finally, "If an EX-SMOKER, how long have you quit smoking for?" Participants were asked to provide details of the number of years and months in order to ascertain whether they had recently quit smoking and may have been smokers during their last hospital admission. Alcohol consumption was assessed using an alcohol frequency table and the "Five Shot Screening Tool" [13], a validated test for detecting hazardous and harmful alcohol use. Participants were asked to provide details of their weight and height, from which we calculated BMI. Daily consumption of 5 portions of fruit and vegetables was taken as an indicator of a healthy diet [2]. Physical activity was assessed by asking how frequently participants undertook 30 minutes of moderate intensity physical activity per week (adapted from the rapid assessment of physical activity scale [14]). Third, information on the type of health promotion delivered within the hospital was gathered. Patients were asked whether they had received leaflets, verbal advice, or referral to a specialist/programme. There was a free text box for describing any additional health promotion delivered. Patients were informed that "by returning the completed questionnaire you are giving you **consent **for your data to be used in this study only". The questionnaire and all related documents received Local Research Ethics approval. The questionnaire is available on request from the authors.

Relevant data abstracted from the case note records and obtained through the patient questionnaire were entered directly into an Excel workbook. The format of the data obtained from the audit and questionnaires was directly comparable. For screening the options were "yes" or "no" for each risk factor. For health promotion delivered the data were transformed into "yes" (some form of health promotion was delivered), "no" (evidence of risk factor, but no health promotion), and "not applicable" (the participant did not have the risk factor and therefore did not require health promotion). For risk factor identified the options were "yes" or "no". Separate researchers collected the audit and questionnaire data. All participants in the audit were designated a unique identification number by one researcher, and this was used on the questionnaire. The researcher inputting data from the questionnaire was blind to the participants' audit data.

## Analysis

Analyses were performed using StatsDirect Version 2.4.5. Patients who returned the questionnaire are described as "responders" and those who did not return the questionnaire "non-responders". Differences in age between responders and non-responders were assessed by one-way ANOVA with gender as a factor. Mann-Whitney U tests were employed to assess differences in length of stay (LoS) between groups. Proportion differences for screening of smoking and alcohol use, smoking behaviour and alcohol misuse (deemed as consuming alcohol above the recommended weekly limits of 21 units for men and 14 units for women) were calculated for responders versus non-responders.

Agreement between the audit and questionnaire findings on screening, identification of risk factors, and health promotion delivered for each risk factor was assessed by simple (unweighted) Kappa statistic. A Kappa statistic below 0.20 was considered as indicative of poor agreement, 0.21 to 0.40 fair agreement, 0.41 to 0.60 moderate agreement, 0.61 to 0.80 good agreement, and 0.81 to 1.00 very good agreement [15].

While we wish to emphasise that we are not assuming that the questionnaire is a superior tool to audit, for ease of understanding the differences between the two sources we have reported the statistics as if this were the case. "Audit False Positives" (AFPs) refers to the proportion of positive audit findings that were negative responses in the questionnaire. "Audit False Negatives" (AFNs) indicates the proportion of negative audit findings that were positive questionnaire responses. Total numbers differ for each agreement analysis as some questionnaires were incomplete and not all patients will engage in the risky behaviour (relevant to each "health promotion needed"). The results of the audit will be reported separately elsewhere.

## Results

Three hundred and twenty two case notes were obtained and audited from a total of 2887 discharges in the whole hospital over the three-month period. Based on this figure, if 50% of the whole patient population received health promotion, we can be 95% confident that the proportion of the patient population who were screened and provided with health promotion is within at least ± 5.15% of the true proportion.

A total of 403 patients were identified. Thirty-two were excluded due to a terminal illness (n = 20) or death (n = 12). Forty-nine were not audited because case notes were not accessible within four weeks of discharge. Patients whose case notes were unattainable were significantly older (mean age of 67.9 ± 1.9 years; two tailed t-test t_(369) _= 4.089 *p *< 0.0001) and had longer lengths of stay than patients whose case notes were audited (median length of stay of 9 days; Mann-Whitney U = 12009, U' = 3769 *p *< 0.0001) (see table 1).

**Table 1:** Demographics for questionnaire responders and non-responders

**AGE**	**Range (years)**	**Total (mean ± SE)**	**Females (mean ± SE)**	**Males (mean ± SE)**
**Responders**	17 – 96	57.0 ± 1.3	56.3 ± 2.0	57.9 ± 1.9
**Non-responders**	17 – 98	52.9 ± 2.0	56.4 ± 3.0	49.4 ± 2.6

**LENGTH OF STAY (LoS)**	**Range (days)**	**Total (median, interquartile range)**	**Females (median, interquartile range)**	**Males (median, interquartile range)**

**Responders**	1 – 50	4 (2–8)	4 (2–8)	5 (2–8)
**Non-responders**	1 – 197	4 (2–10)	4 (1–11)	4 (2–9)

SE: standard error.

One hundred and three females and eighty-seven males returned the questionnaire (59% response rate). The mean age and median length of stay for responders and non-responders are reported in table 1. While male non-responders appear considerably younger than female non-responders and all responders, there was only a trend towards a significant difference in age between the groups (main effect of responder F_(1) _= 3.185, P = 0.08; main effect of gender F_(1) _= 1.279, P = 0.26; responder* gender interaction F_(1) _= 3.328, P = 0.07). There were no significant differences in the median length of stay between any groups. There were no significant differences in the proportion of responders and non-responders who were screened for smoking and alcohol, or identified through case notes as misusing alcohol (see table 2). There were, however, significantly more smokers (according to the audit) amongst non-responders compared to responders.

**Table 2:** Differences in risk factor prevalence between questionnaire responders and non-responders

**Audit findings**	**Responder**	**Non-responder**
Screened for smoking	168/190 (0.88; CI = 0.83 to 0.93)	120/133 (0.90; CI = 0.84 to 0.95)
Screened for alcohol use	145/190 (0.76; CI = 0.70 to 0.82)	101/133 (0.76 CI = 0.68 to 0.83)
Identified as a smoker*	37/167 (0.22; CI = 0.16 to 0.30)	50/119 (0.42; CI = 0.33 to 0.51)
Alcohol consumption above recommendations	26/138 (0.19; CI = 0.13 to 0.26)	25/85 (0.29; CI = 0.20 to 0.40)

*Proportion difference = 0.199, 95% CI = 0.090 to 0.306, P < 0.0005

### Screening

Agreement between the audit and questionnaire for screening of risk factors was at best fair, and at worse poor (see table 3). The number of patients reporting that they were not screened, while their case notes reported that they were (i.e. AFPs), was low for smoking and exercise (in terms of actual numbers rather than proportion) and high in screening for alcohol and diet. The opposite pattern (AFNs) was also evident, with the highest proportion of AFNs for screening of smoking, followed by alcohol, diet, and lastly exercise.

**Table 3:** Agreement between Audit and Questionnaire findings for Screening of risk factors

	Screening Identified			Kappa statistic (95% CI) Strength of agreement	Total Agreement	False positive (95% CI)	False negative (95% CI)
SMOKING							

	Audit						
Questionnaire	Yes	No	Totals				
Yes	143	**13**	156	0.27 (0.13 – 0.41)	83.1%	11.8%	59.1%
No	**18**	9	27	Fair		(7–17%)	(36–79%)

Totals	161	22	183				

ALCOHOL							

	Audit						
Questionnaire	Yes	No	Totals				
Yes	98	**17**	115	0.25 (0.12 – 0.39)	62.2%	31.5%	37.8%
No	**45**	28	73	Fair		(24–40%)	(24–53%)

Totals	143	45	188				

DIET							

	Audit						
Questionnaire	Yes	No	Totals				
Yes	31	**15**	46	0.31 (0.17 – 0.45)	69.0%	57.5%	13.5%
No	**42**	96	138	Fair		(45–69%)	(8–21%)

Totals	73	111	184				

EXERCISE							

	Audit						
Questionnaire	Yes	No	Totals				
Yes	9	**33**	42	0.19 (0.06 – 0.32)	76.6%	47.1%	20.9%
No	**8**	125	133	Poor		(N/A)	(15–28%)

Totals	17	158	175				

*Post hoc *analysis of the AFPs for each risk factor independently was undertaken to determine whether there was any objective evidence that these patients had been screened, but had either forgotten or been unaware that they were screened; and whether lack of recording reflected an absence of the risk factor. According to the questionnaire findings four of the eighteen patients were smokers, however none of the four smokers had any information in their case notes indicating that they were smokers. These latter cases appear to be "definite false positives", indicating that potentially 2% (95% CI = 1–6%) of all positive screenings for smoking reported in the audit are incorrect.

Approximately a quarter of participants (12 out of 45) who disputed being asked about their alcohol intake, despite a record in their case notes, showed evidence of alcohol misuse (according to the five shot score). Three of these twelve did have a record of alcohol misuse in their case notes, revealing that in at least these cases the patients were screened, but did not have any recollection/knowledge of this occurring. For the remaining nine participants, the lack of any recording in their case notes that they misused alcohol suggests that either the quality of the screening process was poor or that it did not happen despite the record. This indicates that potentially 9/143 cases with a screening record in their case notes are wrongly identified as sensible alcohol users when the converse may be true (95% CI = 2.9–11.6%).

For diet, only one patient in the AFPs reported receiving any health promotion for diet (suggesting that she/he was indeed screened). Thirty-four out of the forty-two AFPs reported consuming less than the recommended five portions of fruit and vegetables a day but only eight were deemed as requiring health promotion for diet (again indicating inadequate or absent screening). For exercise, half of the AFPs (4/8) engaged in the recommended amount of physical activity: thirty minutes of moderate exercise five times a week [2], two of whom reported exercise health promotion.

Turning to the data for "Audit False Negatives", *Post hoc *analysis of the smoking AFNs revealed that three of the eighteen were smokers (according to questionnaire findings). For alcohol, three of the seventeen AFNs showed evidence of an alcohol problem (Five shot assessment). For diet, thirteen out of fifteen did not consume the recommended amount of fruit and vegetables. For exercise, twenty-three out of thirty-three did not do enough physical activity.

### Health promotion delivered

Agreement between the audit and questionnaire was no better than chance for smoking and diet (see table 4). The kappa statistic for exercise was again low, but moderate for alcohol. The sample sizes for patients identified by the audit as requiring health promotion were too small for each risk factor to allow statistically meaningful calculation of AFPs. AFNs could be calculated for smoking, but due to the small sample sizes, the 95% confidence intervals were very wide. Approximately 10 to 20 percent of cases that the audit identified as not receiving health promotion for diet and exercise did in fact receive some type of health promotion during their hospital admission (AFNs).

**Table 4:** Agreement between Audit and Questionnaire findings for health promotion delivered

	Health Promotion Delivery Identified			Kappa statistic (95% CI) Strength of agreement	Total agreement	False positive (95% CI)	False negative (95% CI)
SMOKING							

(based on identified need)*	Audit						
Questionnaire	Yes	No	Totals				
Yes	7	**6**	13	NS	65%	61.5%	24%
No	**8**	19	27			(NA)	(9–45%)

Totals	15	25	40				

ALCOHOL							

(based on identified need)*	Audit						
Questionnaire	Yes	No	Totals				
Yes	6	**2**	8	0.50 (0.13 – 0.88)	77.8%	40.0%	11.8%
No	**4**	15	19	Moderate		(NA)	(NA)

Totals	10	17	27				

DIET							

	Audit						
Questionnaire	Yes	No	Totals				
Yes	4	**25**	29	NS	79.8%	71.4%	15.7%
No	**10**	134	144			(NA)	(10–22%)

Totals	14	159	173				

EXERCISE							

	Audit						
Questionnaire	Yes	No	Totals				
Yes	4	**29**	33	0.17 (0.07 – 0.26)	82.6%	20.0%	17.4%
No	**1**	138	139	Poor		(NA)	(12–24%)

Totals	5	167	172				

* Identified need determined by audit findings as opposed to questionnaire findings. NS: P value for Kappa statistic was not significant, indicating that agreement was not different from chance. NA: 95% CI not applicable as sample size too small.

### Risk factor identification

Agreement between the audit and questionnaire for identifying risk factors was very good for obesity, good for smoking, and moderate for alcohol misuse, regardless of whether it was measured by self-reported alcohol units or the "Five shot" tool (see table 5). AFPs were quite low for obesity and smoking, but high for alcohol, with potentially between 12 to 67% of patients wrongly identified as having an alcohol problem. AFNs were fairly low for all risk factors. Again agreement on alcohol misuse was worst, with up to 23% of patients incorrectly diagnosed (according to the Five shot tool) as not having an alcohol problem. The sample sizes for patients identified through the audit as having poor diets (n = 14) and/or physical inactivity (n = 5) were too small to allow statistical comparison with the questionnaire findings.

**Table 5:** Agreement between Audit and Questionnaire findings for risk factor identified as present

	Risk Factor Identified			Kappa statistic (95% CI) Strength of agreement	Total Agreement	False positive (95% CI)	False negative (95% CI)
SMOKING	Audit						

Questionnaire	Yes	No	Totals				
Yes	33 (26)*	**3**	36	0.89 (0.73 – 1.05)	96.2%	8.3%	2.5%
No	3 (9)*	118	121	Good		(2–22%)	(1–7%)

Totals	36	121	157				

ALCOHOL	Audit						

Questionnaire (self report units)	Yes	No	Totals				
Yes	14	**8**	22	0.50 (0.13 – 0.88)	85.4%	46.2%	7.2%
No	**12**	103	115	Moderate		(27–67%)	(3–14%)

Totals	26	111	137				

	Audit						
Five shot	Yes	No	Totals				
Yes	19	**17**	36	0.50 (0.34 – 0.67)	82.6%	26.9%	15.2%
No	**7**	95	102	Moderate		(12–48%)	(9–23%)

Totals	26	112	138				

OBESITY	Audit						

Questionnaire	Yes	No	Totals				
Yes	9	**0**	9	0.82 (0.54 – 1.09)	93.9%	25%	0%
No	**3**	37	40	Very good		(NA)	

Totals	12	37	49				

DIET	Audit						

Questionnaire	Yes	No	Totals				
Yes	13	**12**	25	NS	56.7%	7.1%	75%
No	**1**	4	5			(NA)	(NA)

Totals	14	16	30				

EXERCISE	Audit						

Questionnaire	Yes	No	Totals				
Yes	4	**3**	7	0.48 (0.00 – 0.95)	75.0%	20.0%	27.3%
No	**1**	8	9	Moderate		(NA)	(NA)

Totals	5	11	16				

* Numbers shown are those who have been designated as smokers by the researchers based on the finding that they reported quitting smoking <28 days ago in the questionnaire. Numbers in brackets are the numbers of people for each category when categorised according to self-report of being a current smoker at the time of completing the questionnaire. NS: P value for Kappa statistic was not significant, indicating that agreement was not different from chance. NA: 95% CI not applicable as sample size too small.

## Discussion

The picture that emerges comparing audit findings with patients' recollection is very complex. Looking at the simple agreement (Kappa statistic) between the audit and questionnaire data suggests that neither tool alone is accurate in reflecting the screening and delivery of health promotion, but both are good for risk factor identification. An assessment of the "Audit False Positives" and "Audit False Negatives" may help elucidate where recommendations for change based on audit findings alone are misleading.

It is important to reiterate that the questionnaire is not viewed as a "gold standard" with which to compare audit results as it relies on participants' memories, which are fallible [16,17]. In addition, patients might not answer truthfully for various reasons including social desirability tendencies or demand characteristics of the situation [17]. Direct observation (the "gold standard") of medical services is however not always possible. Apart from the cost and potential ethical objections (some healthcare professionals and patients may object to covert observation once it is revealed, and overt observation may change behaviour), there are practical problems: observing the whole of a patient's hospital episode would be very difficult to achieve as length of stay may last from hours to weeks, involve changing wards, and patient contact with many different healthcare professionals. While audit and questionnaire tools are subject to inaccuracies, by comparing and contrasting the data gathered from both measures, we aimed to provide further insight into health promotion practice within a hospital setting, thereby providing valid information for planning health promotion services and programmes.

### Screening

The substantial discrepancy between the audit and questionnaire findings may reflect the fact that the majority of patients would be asked about risk factors on admission (or pre-admission in the case of elective surgery). Patients may well forget they were asked about risk factors because they were primarily concerned with their condition, and outcome of their hospitalisation. The effect of such distress/anxiety has been shown to lead to attentional narrowing: only the central/most important message(s) is attended to and peripheral information such as treatments, health promotion, etc is not as well attended [18]. Anxiety may also result in state dependent learning: recall of information is most likely when the person is in the same (anxious) state as when the information was imparted [16]. In addition, admission is when patients are often in the most critical condition, and in varying degrees of discomfort/lucidity. The salience of screening questions would also be reduced if the patient did not engage in the risk behaviour. The latter hypothesis is partially supported by the finding that the majority of AFPs were non-smokers and did not misuse alcohol according to their responses on the questionnaire. However most people consumed less fruit and vegetables than is recommended (indicative of a poor diet); and half of the AFPs for exercise screening did not engage in the recommended amount of physical activity. While the proportion of "definite false positives" who were smokers was relatively small, for alcohol, the true proportion of patients that have a false record of alcohol screening in their case notes, and an undiagnosed alcohol problem (based on the Five shot tool), could be as high as 12% of all patients supposedly screened for alcohol use.

The above findings concerning "Audit False Positives" indicate that the results of the audit are to some extent misleading concerning the service that staff are delivering, underestimating the need for changes to improve screening for risk factors. It is also possible that some healthcare professionals are being misled by false information: because hospital staff will wrongly believe that a patient has already been screened and found negative for a risk factor, they may not question a patient further about risk factors, and will therefore miss some patients who do have unhealthy lifestyles. From an epidemiological standpoint prevalence of the risk factor(s) in the patient population will be underestimated. This may also have a negative repercussion on the services offered. For example, if you believe you have *n*% of smokers in the hospitalised patient population but in reality there are more, the smoking cessation services provided by the hospital may be inadequate to meet need and the hospital does not have the "evidence" of a larger smoking population to warrant the commissioning of more services.

The "Audit False Negatives" indicate that patients were in reality screened, but the case notes provided no indication of patients' risk factor status. We hypothesised that AFNs may be indicative of staff's belief that the absence of a risk factor does not need to be noted. While this is supported by the finding that only a small number of the AFNs were smokers or misused alcohol, in the case of diet and exercise, the majority of AFNs did report poor diets and low levels of physical activity respectively. It is of concern that any patients were screened and the finding that they do have a risk factor(s) not recorded. This suggests that some member(s) of hospital staff are aware that a patient is a smoker, misuses alcohol, has a poor diet, etc but no record (or further action) has been taken. While AFNs can be interpreted as indicating that practice is in reality better than the audit results indicate, the fact that there is no record of the outcome of the screening process is a concern in the case of those individuals who are positive for a risk factor(s), and indicates a mismanagement of patients.

### Health promotion delivered

The finding that agreement between the audit and questionnaire was no better than chance for health promotion delivered for smoking and diet, and poor for exercise, is troubling. The small sample size for smoking may be the reason for such poor agreement, but this was not the case for diet or exercise. Actual numbers of "Audit False Positives" for each risk factor were fairly small, as were the number of patients receiving any health promotion, hence it is difficult to draw conclusions concerning the true proportion of AFPs. On the other hand, there are a substantial number of patients who reported receiving health promotion for diet and exercise, but who do not have any evidence of this in their case notes (AFNs). This underreporting of health promotion may result in some unnecessary changes to practice as health promotion delivery is being underestimated. AFNs were fairly low for smoking and very low for alcohol health promotion.

The Ottawa Charter definition of health promotion describes health promotion as a process that enables individuals to change behaviour(s) in order to lead a healthier life, not something which is done to an individual [1]. Hence health promotion aimed at smoking cessation should provide the patient with personalised information and support which enables them to stop smoking (e.g. taking into account the person's motivation to quit, reasons for not wanting to quit, barriers to quitting, etc). This study did not determine the content or the quality of the health promotion services delivered to patients. Written materials, verbal advice and specialist services may be limited to health education or may be "health promoting". The content and quality of these services requires further investigation.

Unlike the situation with screening, if patients can not recall receiving health promotion, then this may be deemed as akin to not receiving it, as it is clearly not enabling change. Lack of recall of health promotion can be influenced by the communication style of healthcare professionals delivering health promotion services [19] and failings in patients' recall [16]. It has been shown that patients are more likely to remember medical information if healthcare professionals provide patients with simple to follow, specific instructions (rather than general instructions) [16]. Memory is also affected by the form of presentation of information: medical information provided verbally is the least likely to be recalled, with adherence to recommendations most likely to be achieved when spoken information is combined with written material (or pictographs in the case of patients with low literacy/education) [16].

Patients memory for health promotion may also be affected by their emotional state when information is imparted (cf. state dependent learning and attentional narrowing), the perceived importance of information (diagnosis is viewed as very important and treatment less so), and age-related cognitive impairments [16]. Recall of the content of health-related information and behaviour recommendations (specifically for alcohol use) has also been shown to be affected by an individual's own beliefs: if the information is in accordance with the person's pre-existing beliefs or health-related practices, it is more likely to be accurately recalled than when the information contradicts one's own beliefs/practices [20]. This latter finding has important ramifications for the delivery of health promotion, indicating that the person delivering health promotion should explore patients' prior beliefs about their health and behaviour and make explicit links between recommendations for change and pre-existing health beliefs [20]. Clearly if hospital healthcare professionals are expected to deliver health promotion, then it is of paramount importance that they are trained to deliver it in a manner which optimises patient recall.

### Identification of risk factor

The agreement between the audit and questionnaire for identification of risk factors was much better than for screening and health promotion delivered. The highest agreement was for obesity, with a very good kappa statistic, no AFNs and few AFPs. This finding probably reflects the fact that obesity, as currently defined, is a relatively objective measure, reliant on measurements of weight and height, while all other risk factors are reliant on the truthfulness of the patient answering questions. Complexity of screening is also an issue – body mass index (BMI) is a simple measurement that does not necessarily require staff to make a calculation as BMI charts are available. In this particular instance, the audit tool may be seen as the "gold standard" compared to self-reported height and weight as patients may underreport weight [21]. However the results reported in table 5 indicate that respondents were fairly truthful about their weight, with only 3 people reporting a weight indicative of overweight, while their case notes classified them as obese (and this difference in weight categories may be due to the participants losing weight).

Smoking, the risk factor with the next best agreement between the audit and questionnaire, is also simple to assess, and can be achieved with one question. Alcohol is more difficult to assess, with self-report of alcohol consumption subject to memory loss and misleading information. Staff may have difficulty precisely calculating alcohol units based on a patient's verbal description of alcohol consumed. Stigmatism may play a part in how well staff can assess a risk factor, with patients possibly feeling that smoking and (probably more so) alcohol are deemed by staff as unacceptable behaviours and self-inflicted. The identification of poor diet and low levels of physical activity have not been subject to agreement analysis because this type of information is rarely recorded, in part probably due to the fact that there are no quick/simple questions to assess regular diet or exercise.

Identification of alcohol misuse fared the worst out of all the risk factors. Within the hospital no validated screening tool for alcohol consumption is currently recommended or routinely used for evaluating alcohol misuse, and the general rule is to verbally ask people about their weekly alcohol consumption. The latter method of alcohol consumption assessment is subject to error due to staff not knowing recommended alcohol limits and/or not knowing how many units are contained in different types of alcohol. The main concern for risk factor identification is AFNs": people identified as not having a "problem" when the converse is true. The percentage of AFNs when the Five shot tool was used was approximately twice as high as when the audit findings were compared to patients' self-reported units (questionnaire). Based on the five shot tool findings, between 9% and 23% of patients may be misdiagnosed as not having an alcohol problem. These findings support previous recommendations that validated alcohol screening tools are more appropriate for detecting alcohol misuse compared to relying on self-reported alcohol consumption [22].

## Conclusion

Our study indicates that audit alone may not accurately reflect health promotion practice within hospitals. We therefore make the following recommendations for the monitoring of health promotion practice within hospitals:

• A combination of audit and questionnaire is appropriate in identifying the proportion of patients who are screened for a risk factor. Audit results can be viewed as accurate except when those with a screening "Audit False Positive" also have an "Audit False Negative" for risk factor identification. These cases indicate that the audit results are overestimating the proportion of patients screened and/or the accuracy of screening. Secondly, it is worth determining the screening "Audit False Negatives" as these cases indicate that practice is better than the audit results imply.

• In the case of health promotion delivered self-report (questionnaire) is the preferred method.

• The good agreement between the audit and questionnaire for identifying risk behaviours indicates that either method is appropriate. However in hospitals which rely on self-reported alcohol intake as a measure for alcohol misuse, the questionnaire is superior as it includes a validated screening tool.

The general methodology of supplementing audits with patient questionnaires has proven both useful and workable, and can be generalised to other clinical areas. However, the requirement to send a questionnaire within one month of a patient's discharge may mean the exclusion of patients who are older and who have long lengths of stay (as indicated in the demographics of patients whose case notes were unobtainable). Practitioners may also be reluctant to undertake such a study, as audit alone has the advantage of not requiring ethics approval and access to information from a large sample size. The development of a questionnaire to compare with an audit takes time and ethics approval. In addition, the sample size from a questionnaire may be too small to allow statistical analysis, although in this study the response rate was good.

The sample size was however an issue in the number of patients identified as smokers and/or identified as misusing alcohol. The small sample sizes meant statistical analysis of the agreement between written case notes and patients' self-report of health promotion delivered was limited. While we would suggest repeating this study with a larger sample of smokers and hazardous/harmful drinkers, the difficulty lies in knowing *a priori *whether someone is a smoker or misuses alcohol. As the methodology relies on using a random sample of case notes, the only way to guarantee a larger sample size of smokers/alcohol misusers is to audit a larger sample of case notes. Within a hospitalised population we would expect 20% of men and 10% of women to consume an amount of alcohol exceeding internationally recommended limits [23].

While there is evidence that non-responders were more likely to be smokers than responders, the prevalence of smoking in responders in this study is identical to the prevalence in the general population of Stockport (22%) [24], suggesting that smokers are not necessarily avoiding completing the questionnaire (or alternatively, that surveys in general are underreporting the true prevalence of smoking in the community). In line with the small sample size of patients identified through the audit as having a risk factor, because so few patients also receive health promotion, agreement analysis for health promotion delivered was statistically limited.

While there are difficulties inherent in replicating this study in other clinical disciplines, the finding that there are substantial inconsistencies between what is written in a patient's case notes and what (according to the patient) has occurred, indicates that this type of work should be undertaken in other disciplines which do not have well-established protocols and integrated care pathways. This study was undertaken in only one hospital and findings may therefore not be generalisable to health promotion practice within other hospitals. Implementing this methodology in other hospitals will provide valuable information on the quality of data in patients' written case notes; and provide hospitals with data to support the indicators in standards two and three of the WHO health promotion hospitals network [5].

We suggest that the quality of health promotion documentation may be improved if hospitals implemented clear guidelines concerning how to identify and "treat" risk factors. This would include the introduction of validated screening tools for alcohol, diet, and exercise and simple integrated care pathways providing healthcare professionals with guidelines on referral processes and the actions that can be taken to deliver appropriate health promotion.

## Competing interests

The author(s) declare that they have no competing interests.

## Authors' contributions

CH and GC made substantial intellectual contributions to this study. Both were involved in the conception and design of the project. CH along with a research assistant collected the data. CH analysed the data. CH interpreted the data. CH wrote the bulk of this article, which was then revised by GC. Both authors have read and approved the final manuscript.

## Pre-publication history

The pre-publication history for this paper can be accessed here:


